# A comparison of contiguous two-level anterior cervical discectomy and fusion using a structural allograft versus a Polyetheretherketone (PEEK) cage: the results of a three-year follow-up

**DOI:** 10.1186/s12891-020-03325-y

**Published:** 2020-05-28

**Authors:** Ing How Moo, Carmen Jia Wen Kam, Maksim Wen Sheng Lai, William Yeo, Reuben Chee Cheong Soh

**Affiliations:** 1 Department of Orthopedic Surgery, Singapore General Hospital, Outram Road, Singapore, 169608 Singapore; 2 Changi General Hospital, 2 Simei Street 3, Bukit Merah, 529889 Singapore

**Keywords:** Cervical, ACDF, Allograft, Polyetheretherketone, PEEK

## Abstract

**Background:**

Allografts and polyetheretherketone (PEEK) cages are the two most commonly used materials in anterior cervical discectomy and fusion (ACDF). However, their effectiveness in two-level ACDF remains controversial. The primary aim of this retrospective study was to compare the clinical and radiological outcomes of two-level ACDF with plate fixation using either a structural allograft or a PEEK cage.

**Methods:**

From 2010 to 2015, 88 consecutive patients underwent two-level ACDF, of whom 53 received an allograft and 35 patients received a PEEK cage. All PEEK cages were filled with local autografts. All clinical outcomes were prospectively collected before and six months and two years after surgery. Clinical efficacy was evaluated using a visual analogue scale for neck pain and limb pain, the Neck Pain and Disability Score, the Neck Disability Index, the Neurogenic Symptom Score, and the Japanese Orthopedic Association score. Radiological outcomes were assessed preoperatively, immediately after surgery, and at the final follow-up.

**Results:**

A preoperative comparison revealed no difference between the two patient groups in terms of age, sex, body mass index, smoking status, preoperative symptoms, operation level, or follow-up (mean = 42.8 months). No differences in the improvements in clinical outcomes were observed between the two groups. Both groups showed significant improvement in mean disc height, segmental height, and segmental lordosis postoperatively. The fusion rate for the PEEK cage was 100% at both levels, while the fusion rate for the allograft group was 98.1% at the cephalad level and 94.2% at the caudad level (*p* > 0.05). Subsidence at the cephalad level occurred in 22.9% (8/35) of segments in the PEEK group and 7.7% (4/52) of segments in the allograft group (*p* = 0.057). At the caudal level, a higher incidence of cage subsidence was noted in the PEEK group than in the allograft group [37.1% (13/35) versus 15.4% (8/52)] (*p* = 0.02). Overall, subsidence was noted in 30% (21/70) of the PEEK group and in 11% (12/104) of the allograft group (*p* <  0.05).

**Conclusion:**

The use of PEEK cages resulted in a higher rate of subsidence in two-level ACDF than the use of allografts. Two-level ACDF using either allografts or PEEK cages resulted in similar clinical outcomes, radiological improvements in alignment and fusion rates.

## Background

Anterior cervical decompression and fusion (ACDF) was first described by Smith and Robinson in 1955 and has since become a highly effective procedure to treat degenerative cervical disc disease [1]. However, the success rate declines for multilevel ACDF because contact stress and micromotion increase at the graft–body interface, which may affect fusion as well as maintenance of the height of the neural foramen [2].

The ideal interbody graft material for this procedure remains to be determined. The use of an autologous iliac crest (AIC) bone graft is considered by many to be the gold standard to achieve a high interbody fusion rate. However, AIC harvesting has a 13% donor site complication rate [3, 4]. This is the impetus to seek several different biomaterials that can allow for maintenance of the disc height and subsequent fusion across the interbody space.

Allografts and polyetheretherketone (PEEK) cages are the two most commonly used materials in ACDF and comprise 92% of interbody cages [5]. Since their commercial release in 1998, PEEK cages have been widely used despite a lack of evidence on their outcomes compared with those of allograft interbody cages [6].

To the best of the authors’ knowledge, there are no studies that directly compare the outcomes of PEEK cages versus those of allografts in two-level contiguous ACDF. The primary aim of this study was to evaluate the long-term clinical efficacy and radiological outcomes of two-level ACDF with plate fixation using either a structural allograft or a PEEK cage.

## Methods

Approval was obtained from the Centralized Institutional Review Board of Singhealth (CRIB: 2017/2628). All patients who underwent two-level contiguous ACDF surgery with plating using either PEEK cages (Cornerstone®, Medtronic Sofamor-Danek, Memphis, TN, USA; Cervios®, Synthes, Zuchwil, Switzerland; Solis® Stryker Spine, Allendale, NJ) or allografts (Triad® Allograft system, Nuvasive, Inc.) from 2010 to 2015 were selected. The allograft used was a saline-packaged femoral or tibial cortical–cancellous allograft with 7^▯^ of lordosis that was terminally sterilised with low-dose irradiation. The allograft was precision-machined on all sides, has similar dimensions to the PEEK cages and is ready for use intraoperatively. For this study, only patients who underwent surgery for degenerative cervical disc disease and spondylosis and who had a minimum two-year follow-up were included. The indications for surgery were symptomatic cervical radiculopathy, myelopathy or myelo-radiculopathy with demonstrably correlated two-level compression based on preoperative magnetic resonance imaging (MRI). Patients with peripheral neuropathy, parkinsonism, psychiatric illness, tumours, fractures, previous cervical spine surgery, a standalone cage, or infections were excluded.

All surgeries were performed in a single centre by orthopaedic spine surgeons using standard operative techniques as described. A Smith–Robinson approach to the anterior cervical spine was performed. Once the operative level was identified radiologically, Caspar pins and retractors were used to gain exposure. Microsurgical decompression was then performed extending laterally to the uncinate processes with partial removal of the posterior uncinate process to free the neural elements. The posterior longitudinal ligament was subsequently resected. Endplate preparation involved the use of a high-speed burr to remove the overlying cartilage up to the bleeding subchondral bone. An optimal size interbody material was selected and inserted into the disc space. Either a PEEK cage or an allograft was used depending on the surgeon’s preference. All PEEK cages were filled with local autografts from anterior osteophytes as well as shavings from burring the uncinate process. A cervical plate and screws were used for fixation in all cases of ACDF. All cages were lordotic in nature. The sizes used were based on intraoperative sizing and ranged from 5 to 7 mm. An Aspen cervical collar was used for 6 weeks postoperatively. All patients were managed postoperatively according to our institution’s cervical spinal surgery clinical pathway, and all patients underwent the same physiotherapy protocol.

### Outcomes assessment

Demographics, perioperative details, and clinical outcomes were independently collected at our institution’s Orthopaedic Diagnostic Centre, which evaluates all patients undergoing spine surgery preoperatively and postoperatively at 6 months and 2 years. Next, a retrospective analysis of the data was performed. The following outcome scales were used: the Neck Pain and Disability (NPD) scale, American Academy of Orthopaedic Surgeons Neurogenic Symptom Score (AAOS-NSS), the Neck Disability Index (NDI), visual analogue scale for neck pain (VASNP), visual analogue scale for limb pain (VASLP), and Japanese Orthopedic Association (JOA) score.

**Fig. 1:**
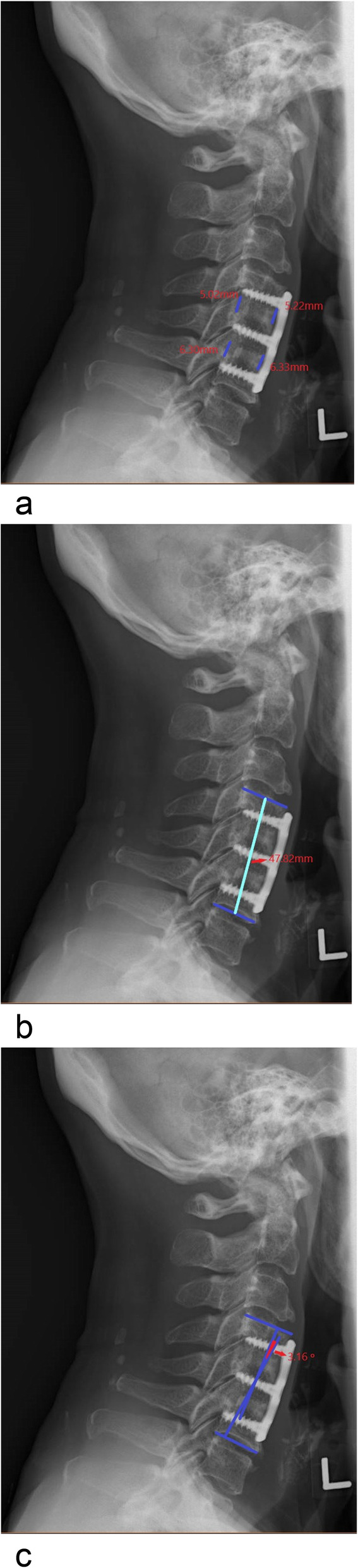
**a** The method for measuring disc height: the mean value of the anterior and posterior disc height at each level; **b** the method for measuring segmental height: the distance between the middle margin of the upper end plate of the superior vertebral body and the lower end plate of the inferior vertebral body; **c** the method for measuring segmental Cobb angle/lordosis: the angle formed by the upper endplate of the superior vertebrae body and the lower endplate of the inferior vertebrae body, while the patient is in a neutral position

Radiographs were taken before surgery, immediately after surgery, and at the last follow-up. Digital radiographs stored in the Picture Archiving and Communication System were used to measure distances and angles with accuracies up to 0.01 mm and 0.1 degrees, respectively. The radiological parameters assessed for each level include mean disc height, segmental height, segmental Cobb angle, and the C2-C7 Cobb angle (Fig. 1). Subsidence was defined as the loss of more than 2 mm of segmental height at the final follow-up compared to the segmental height measured immediately after surgery. In accordance with current evidence, fusion was defined by the following factors: 1) the interspinous distance (lack of movement at the operated levels with interspinous process motion having a < 1 mm difference in flexion and extension on an adequate scan, which was defined as the presence of interspinous process motion of at least 4 mm at the uninvolved adjacent segment), 2) the presence of a bridging bone across the fusion level observed on a computed tomography (CT) scan or a plain radiograph at the last follow-up, and 3) the absence of radiolucency at the graft–vertebral junction [7]. There were no patients in our study with postoperative infection or who required reoperation within an average of 3 years of follow-up.

### Statistical analysis

Statistical analysis was performed by a statistician using SPSS statistical software, version 19.0 (IBM Corp. Armonk, NY).

With an expected ratio of 1.5 of the number of PEEK cages to the number of structural allografts applied and a hypothesized medium size effect (Cohen’s d = 0.6) between the two groups on outcomes, for a power of 80% and a level of significance of 5% (two sided), the number of samples required was at least 37 for the PEEK group and 56 for the allograft group. Our study included 35 patients in the PEEK group and 53 patients in the allograft group. Therefore, our study was slightly underpowered.

Categorical data are presented as frequency (percentage) and were examined using a chi-squared test. Numerical data following a parametric distribution are presented as the mean ± standard deviation and those following a nonparametric distribution are presented as the median (interquartile range). A two-sample t-test was used to evaluate the numerical variables. For clinical and radiological outcomes, a two-way repeated measures ANOVA was used to examine the significance of the group*time interaction and the main effects of groups and time. If there was a significant group*time interaction, a subgroup analysis of groups and time was performed, and the simple main effects of group type and time were tested independently. If there was no significant interaction, the main effects of protocol type and time were reported. A two-tailed *p*-value of < 0.05 was considered statistically significant.

## Results

Eighty-eight cases of two-level ACDF with either allografts (*n* = 53) or PEEK cages (*n* = 35) matched the strict inclusion criteria. A preoperative comparison showed that there was no significant difference between patients who received PEEK cages versus allografts in terms of age, sex, body mass index, smoking status, preoperative symptoms, operated levels, and preoperative scores according to the NPD scale, AAOS-NSS, NDI, VASNP, and VASLP (*p* > 0.05) (Table 1). The allograft group had a higher JOA score at baseline than the PEEK group. Both groups had postoperative follow-up periods of similar duration (mean = 42.8 months, ranging from 24 to 58 months). No instances of complications were reported in either group.

**Table 1:** Preoperative comparison between Allograft and PEEK groups

**Demographics**	**Allograft** **(*n*** **= 53)**	**PEEK** **(*n*** **= 35)**	***p*-value**
Male	27 (50.9)	19 (54.3)	0.759
Age (years)	52.4	56.0	
Median (IQR)	(45.2–62.1)	(48.2–63.2)	0.188
BMI	25.0	25.0	
Median (IQR)	(22.7–27.1)	(23.1–28.2)	0.617
Current smoker	6 (11.5)	4 (11.8)	1.000
Symptom			0.335
Radiculopathy	20 (37.7)	8 (22.9)	
Myelopathy	20 (37.7)	17 (48.6)	
Radiculomyelopathy	13 (24.5)	10 (28.6)	
Level			0.141
C3–4/C4–5	3 (5.7)	5 (14.3)	
C4–5/C5–6	14 (26.4)	12 (34.3)	
C5–6/C6–7	36 (67.9)	16 (45.7)	
C3–4/C5–6^*^	0 (0.0)	1 (2.9)	
C4–5/C6–7^*^	0 (0.0)	1 (2.9)	
**Clinical baseline**			
NPD	31.3 ± 20.0	39.9 ± 26.8	0.111
AAOS-NSS	47.7 ± 25.9	49.6 ± 29.8	0.746
NDI	30.3 ± 20.6	38.9 ± 25.1	0.083
VASNP	4.8 ± 3.5	4.6 ± 3.4	0.751
VASLP	4.1 ± 3.9	2.7 ± 3.2	0.067
JOA	13.7 ± 1.9	11.3 ± 4.3	0.008
**Radiology baseline**			
Cervical cobb angle	7.24 ± 11.97	10.96 ± 9.80	0.130
**Cephalic level**			
Mean Disc Height	3.47 ± 1.07	3.97 ± 1.25	0.048
Segmental height	31.32 ± 3.66	31.57 ± 4.11	0.769
Segmental Cobb angle	−1.19 ± 5.83	2.30 ± 6.66	0.011
**Caudad level**			
Mean Disc Height	3.58 ± 1.09	3.76 ± 1.74	0.575
Segmental height	32.27 ± 3.51	31.10 ± 3.41	0.125
Segmental Cobb angle	3.41 ± 4.77	2.25 ± 6.06	0.322

^*^Small frequency – excluded in the calculation of *p*-value

*NPD* Neck pain and disability; *AAOS-NSS* AAOS neurogenic symptom score; *NDI* Neck Disability Index; *VASNP* Visual analog score neck pain; *VASLP* Visual analog score limp pain; *JOA* Japanese Orthopedic Association Score;

At 6 months and 2 years postoperatively, both groups demonstrated significant improvements in NPD score, NSS, NDI, JOA score, VASNP, and VASLP, but there were no significant differences between the two groups (Table 2). Although both groups had similar JOA scores at 6 months postoperatively (*p* = 0.137), the allograft group had a higher JOA score at 2 years postoperatively than the PEEK group (*p* = 0.03).

**Table 2:** Clinical outcomes between the allograft and PEEK group

							Baseline versus 6-month			Baseline versus 2-year		
Clinical outcome	Allograft (*n* = 53)			PEEK (*n* = 35)			Interaction between Group and Time	Group	Time	Interaction between Group and Time	Group	Time
	Preoperative	6-month	2-year	Preoperative	6-month	2-year	*p*-value	*p*-value	*p*-value	*p*-value	*p*-value	*p*-value
NPD	31.2 ± 20.3	14.0 ± 16.9	8.8 ± 12.7	39.7 ± 27.2	20.6 ± 17.4	13.4 ± 13.8	0.667	0.063	< 0.001	0.365	0.052	< 0.001
NS	47.3 ± 26.1	15.7 ± 22.3	14.6 ± 21.9	50.4 ± 29.9	18.2 ± 18.8	12.8 ± 14.0	0.928	0.543	< 0.001	0.649	0.929	< 0.001
NDI	30.4 ± 20.9	15.1 ± 16.1	10.4 ± 15.1	38.5 ± 25.4	18.5 ± 17.3	11.4 ± 11.9	0.261	0.145	< 0.001	0.126	0.174	< 0.001
VASNP	4.8 ± 3.6	1.3 ± 2.5	1.0 ± 2.3	4.4 ± 3.4	1.9 ± 3.0	0.9 ± 2.3	0.260	0.778	< 0.001	0.939	0.832	< 0.001
VASLP	4.1 ± 3.9	0.8 ± 2.0	0.9 ± 2.2	2.7 ± 3.2	0.5 ± 1.7	0.2 ± 1.2	0.129	0.107	< 0.001	0.451	0.087	< 0.001
JOA	13.7 ± 2.0	15.3 ± 2.1	15.5 ± 2.1	11.3 ± 4.3	14.3 ± 3.5	14.3 ± 3.2	0.011	0.162	< 0.001	0.156	0.030	< 0.001

Mean ± SD. Two-way Repeated Measures ANOVA. Interaction and main effects of groups and time of measurement were assessed. Significance level was set at *p* <  0.05

*ROM* Range of motion; *NPD* Neck pain and disability; *AAOS-NS* AAOS neurogenic symptom score; *NDI* Neck Disability Index; *VASNP* Visual analog score neck pain; *VASLP* Visual analog score limp pain; *JOA* Japanese Orthopedic Association Score;

At both operated levels, both groups demonstrated significant improvement in mean disc height and segmental height immediately after surgery. Loss of mean disc height and segmental height were noted in both groups at both levels at the final follow-up. There were no differences in mean disc height and segmental height (*p* > 0.05) between the two groups immediately after surgery or at the last follow-up (Table 3).

**Table 3:** Radiological outcomes between the allograft and PEEK groups

Cephalad level							Pre-op versus first post-op			First post-op versus final post-op		
Radiological Outcome	Allograft (*n* = 53)			PEEK (*n* = 35)			Interaction (Group*Time)	Group	Time	Interaction (Group*Time)	Group	Time
	Preop	1st post-op	Final postop	Preop	1st post-op	Final postop	*p*-value	*p*-value	*p*-value	*p*-value	*p*-value	*p*-value
Mean Disc Height	3.47 ± 1.07	6.51 ± 0.89	5.46 ± 0.89	3.97 ± 1.25	6.63 ± 0.98	5.64 ± 0.91	0.173	0.088	< 0.001	0.848	0.327	< 0.001
Segmental Height	31.32 ± 3.66	33.23 ± 3.44	32.66 ± 3.41	31.57 ± 4.11	33.50 ± 3.69	32.14 ± 3.46	0.947	0.741	< 0.001	0.002	0.490	*0.004 ^< 0.001
Segmental Cobb Angle	−1.19 ± 5.83	1.89 ± 3.82	0.46 ± 4.19	2.30 ± 6.66	2.53 ± 4.52	1.20 ± 3.94	0.034	0.477	*< 0.001 ^0.852	0.954	0.361	0.004
Cervical Cobb Angle	7.24 ± 11.97	11.75 ± 10.41	11.37 ± 9.80	10.96 ± 9.80	10.46 ± 7.80	11.02 ± 8.03	0.014	0.532	*< 0.001 ^0.759	0.519	0.623	0.999
**Caudal level**							Pre-op versus first post-op			First post-op versus final post-op		
Radiological Outcome	Triad (*n* = 53)			Peek (*n* = 35)			Interaction (Group*Time)	Group	Time	Interaction (Group*Time)	Group	Time
	Preop	1st post-op	Final postop	Preop	1st post-op	Final postop	*p*-value	*p*-value	*p*-value	*p*-value	*p*-value	*p*-value
Mean Disc Height	3.58 ± 1.09	6.51 ± 1.09	5.40 ± 0.92	3.76 ± 1.74	6.57 ± 1.06	5.72 ± 1.11	0.680	0.581	< 0.001	0.384	0.272	< 0.001
Segmental height	32.27 ± 3.51	33.94 ± 3.43	33.05 ± 3.41	31.10 ± 3.41	33.14 ± 3.50	31.65 ± 2.94	0.340	0.292	< 0.001	0.017	0.050	< 0.001
Segmental Cobb angle	3.41 ± 4.77	6.95 ± 4.38	4.00 ± 4.92	2.25 ± 6.06	6.04 ± 4.74	3.25 ± 5.26	0.811	0.280	< 0.001	0.895	0.376	< 0.001

Mean ± SD. Two-way Repeated Measures ANOVA. Interaction and main effects of groups and time of measurement were assessed. Significance level was set at *p* < 0.05

*Allograft

^PEEK

Preop, preoperative; post-op, postoperation,

At the cephalad level, the PEEK group had greater segmental lordosis than the allograft group before surgery (*p* = 0.011) (Table 1). Immediately after surgery, segmental lordosis remained unchanged in the PEEK group (*p* = 0.852), while the allograft group demonstrated significant improvement at the cephalad level (*p* <  0.001). At the caudal level, both groups demonstrated significant improvement in segmental lordosis immediately after surgery (*p* <  0.001). There was no difference in the segmental Cobb angle between the two groups at either level immediately after surgery or at the final follow-up (*p* > 0.05). Both groups demonstrated significant loss of the segmental Cobb angle at both levels at the final follow-up (*p* <  0.004).

Both groups had similar cervical Cobb angles at baseline and immediately after surgery (*p* = 0.130 and *p* = 0.532, respectively). At the last follow-up, cervical lordosis was maintained in both groups; there was no difference between the groups (*p* > 0.05).

Concerning cage subsidence, subsidence at the cephalad level occurred in 22.9% (8/35) of the segments in the PEEK group and 7.7% (4/52) of the segments in the allograft group (*p* = 0.057). At the caudal level, a higher incidence of cage subsidence was noted in the PEEK group than in the allograft group [37.1% (13/35) versus 15.4% (8/52)] (*p* = 0.02) (Table 4). Overall, the subsidence rates were 30% (21/70) and 11% (12/104) in the PEEK and allograft groups, respectively (*p* <  0.05).

**Table 4:** Fusion and subsidence rate between the allograft and PEEK groups

	Allograft (*n* = 53)	Peek (*n* = 35)	*p*-value
**Cephalad level**			
Fusion	51 (98.1)	35 (100)	1.000
Subsidence	4 (7.7)	8 (22.9)	0.057
**Caudal level**			
Fusion	49 (94.2)	35 (100)	0.270
Subsidence	8 (15.4)	13 (37.1)	0.020

At the last follow-up, 55 patients underwent fusion assessment using flexion-extension radiographs and the interspinous process criteria (< 1 mm motion difference), 24 patients were assessed using CT scans, and the remaining 9 patients were assessed using static radiographs. There was no significant difference in the fusion assessment methods between the two groups. The fusion rate for PEEK cages was 100% at both levels. The fusion rate for the allograft group was 98.1% at the cephalad level and 94.2% at the caudal level (overall 96.2%). There was no significant difference in the union rate between the two groups (*p* > 0.05) (Table 4). All non-union patients were non-smokers. Reoperation for non-fusion was not needed.

## Discussion

In general, the fusion rate decreases as the number of operative levels increases [8–10]. This study directly compared the clinical and radiological outcomes of allografts and PEEK cages with 2 similar groups who underwent two-level ACDF with anterior cervical plating. This study demonstrated 100 and 96.2% fusion rates for PEEK cages and allografts, respectively.

There was a demonstrable improvement in lordosis in both the allograft and PEEK groups. However, concerning cage subsidence, it was noted that PEEK cages caused more subsidence than allograft cages. Several reasons may help to explain this. First, although PEEK mimics the elastic modulus of bone, this material is non-resorbable and may result in point loading. Second, there is emerging evidence on the presence of fibrous tissues on the bone–implant interface [11], which can slow osteointegration, leading to higher micromotion. In vitro studies have also demonstrated that the osteoblastic differentiation of progenitor cells is reduced on the surface of PEEK cages and that inflammatory chemokines are produced, which may theoretically contribute to subsidence [12]. The literature reported a widely ranging subsidence rate in ACDF for both allografts and PEEK cages (5–43% vs. 8–32%) [5]. Yson et al. compared the subsidence rates between PEEK cages and allografts and found no significant difference between the PEEK cages (29%) and the allografts (28%) [13]. This study included mixed levels of ACDF and defined subsidence using a different criterion.

To date, comparative studies of PEEK cages and allografts in ACDF are of low quality and are heterogeneous. Due to the different cervical biomechanics in single-level versus two-level ACDF, we chose to study only patients with contiguous two-level ACDF [14]. Vaidya et al. performed a retrospective study of 46 patients who underwent ACDF with anterior plating [15]. PEEK cages filled with recombinant human bone morphogenetic protein-2 (rhBMP-2) (*n* = 22, 8 one-level, 9 two-level, 4 three-level) were compared with allograft spacers and demineralized bone matrices (*n* = 24, 11 one-level, 10 two-level, 3 three-level). All patients in the PEEK group achieved union, while 23/24 patients in the allograft group achieved union. The study concluded that there was no difference in the arthrodesis rate between the two groups. However, this study included a mix of single-level and multilevel ACDF, and the use of RhBMP-2 in the PEEK group introduced confounding bias in the analysis of the fusion rate between the two groups. Hence, we sought to look at the fusion rate in patients without the use of BMP. Katie et al. retrospectively reviewed 127 cases of single-level ACDF with either PEEK cages or allografts [16]. Of the patients involved, 29/56 (52%) with PEEK cages had pseudarthrosis compared to 7/71 (10%) patients with allografts. The author concluded that the use of PEEK cages is associated with an increased incidence of non-union and revision surgery compared to the use of structural allografts. However, there are several confounders that affected the validity of this conclusion, including a 69% loss to follow-up rate, more smokers in the PEEK group and the use of various types of allografts. In addition, 82% of the PEEK implants were stand-alone devices, while 100% of the allograft group had anterior cervical plating. Most recently, Pirkle et al. performed a comparative registry study of 6130 patients with ACDF using either allografts or intervertebral cages [17]. Non-union was identified by coding, and the analysis included only 3 variables (i.e., smoking, diabetes and operation level). The study concluded that the cage group had a higher non-union rate than the allograft group. However, there were no demographic data, radiographic analysis of fusion or details of the types of cages placed.

In our study, the fusion rate of 96.2% for allograft cages compares favourably with the results in the literature. Allograft cages with anterior cervical plating have previously shown fusion rates ranging from 92 to 100% [4, 18]. Different types of allografts may also explain the wide range of fusion rates. Allografts vary in bone quality depending on the donor population and the type of bone harvested. In addition, the final biomechanical properties of a particular allograft are significantly influenced by its method of preparation, which may vary widely among manufacturers [19]. The most commonly used allografts are freeze-dried, undergo high-dose irradiation, and include cortical allografts. Cortical allografts lack the three-dimensional bone matrix and have a slower graft incorporation rate than cancellous allografts. However, cortical allografts are less likely to collapse. Freeze-drying, especially in conjunction with irradiation, can cause a significant reduction in strength. All the allografts in our current study were obtained from the same manufacturer and come as saline-packaged cortical–cancellous allografts obtained from human femurs or tibias and were terminally sterilised with low-dose irradiation to maintain mechanical integrity.

Maintaining cervical disc height after surgery is crucial, as disc height collapse may result in kyphosis formation and accelerate adjacent segment degeneration in the long term [13]. In our study, patients in both groups showed significant improvement in mean disc height, segmental height, and segmental lordosis postoperatively. However, there was a loss of mean disc height, segmental height, and segmental lordosis at the last follow-up for both groups compared to those immediately after surgery. An average of 20% loss in height at each interspace level can be expected, even after tricortical autograft fusion [4].

Our study has certain limitations, such as its retrospective nature, small sample size, and operations performed by multiple surgeons. In addition, the relationship between bone density and cage subsidence was not analysed. The dimensional aspects of the allograft and the PEEK cage in relation to subsidence and fusion rate were also not evaluated in our study. The endplate margin of the vertebrae might not be well defined, and the potential measurement error must also be taken into account. It is difficult to accurately evaluate bone bridge formation and assess dynamic motion on lateral radiographs, and CT scans may not be possible in all cases. The strengths of our study include using a uniform single type of allograft, objective clinical data with validated outcome surveys, and strict criteria for subsidence and fusion, providing the longest follow-up to date on 2-level ACDF cases in the literature, and addressing the lack of previous head-to-head comparisons of the outcomes of PEEK cages and allografts in two-level ACDF.

Furthermore, our study also demonstrated that subsidence does not impact clinical outcomes, which is consistent with the literature.

## Conclusion

While two-level ACDF using either allograft or PEEK cages resulted in similar clinical outcomes and fusion rates, the subsidence rate was higher with the use of PEEK cages.

## Data Availability

The datasets used and/or analysed during the current study are available from the corresponding author upon reasonable request.

## Abbreviations

ACDF: Anterior cervical discectomy and fusion
PEEK: Polyetheretherketone
AIC: Autologous iliac crest
MRI: Magnetic resonance imaging
NPD: Neck pain and disability scale
AAOS-NSS: American academy of orthopaedic surgeons neurogenic symptom score
NDI: Neck Disability Index
VASNP: Visual analogue scale for neck pain
VASLP: Visual analogue scale for limb pain
JOA: Japanese orthopedic association
CT: Computed tomography
rhBMP-2: Recombinant human bone morphogenetic protein-2
